# Forecasting Flu Activity in the United States: Benchmarking an Endemic-Epidemic Beta Model

**DOI:** 10.3390/ijerph17041381

**Published:** 2020-02-21

**Authors:** Junyi Lu, Sebastian Meyer

**Affiliations:** Institute of Medical Informatics, Biometry, and Epidemiology, Friedrich-Alexander-Universität Erlangen-Nürnberg, 91054 Erlangen, Germany; junyi.lu@fau.de

**Keywords:** influenza, forecasting, time series, beta regression, seasonality

## Abstract

Accurate prediction of flu activity enables health officials to plan disease prevention and allocate treatment resources. A promising forecasting approach is to adapt the well-established endemic-epidemic modeling framework to time series of infectious disease proportions. Using U.S. influenza-like illness surveillance data over 18 seasons, we assessed probabilistic forecasts of this new beta autoregressive model with proper scoring rules. Other readily available forecasting tools were used for comparison, including Prophet, (S)ARIMA and kernel conditional density estimation (KCDE). Short-term flu activity was equally well predicted up to four weeks ahead by the beta model with four autoregressive lags and by KCDE; however, the beta model runs much faster. Non-dynamic Prophet scored worst. Relative performance differed for seasonal peak prediction. Prophet produced the best peak intensity forecasts in seasons with standard epidemic curves; otherwise, KCDE outperformed all other methods. Peak timing was best predicted by SARIMA, KCDE or the beta model, depending on the season. The best overall performance when predicting peak timing and intensity was achieved by KCDE. Only KCDE and naive historical forecasts consistently outperformed the equal-bin reference approach for all test seasons. We conclude that the endemic-epidemic beta model is a performant and easy-to-implement tool to forecast flu activity a few weeks ahead. Real-time forecasting of the seasonal peak, however, should consider outputs of multiple models simultaneously, weighing their usefulness as the season progresses.

## 1. Introduction

Influenza is a contagious respiratory illness caused by different types of influenza viruses. The outcomes of flu infections vary widely, and serious infections can cause hospitalization or death. The Centers for Disease Control and Prevention (CDC) in the U.S. estimated that around 8% of the U.S. population becomes infected with influenza during an average season [1]. Accurate prediction of flu activity provides health officials with valuable information to plan disease prevention and allocate treatment resources. Since 2013, CDC organizes the “Predict the Influenza Season Challenge” (https://predict.cdc.gov/, also known as the CDC FluSight challenge) for every flu season, to encourage academic and private industry researchers to forecast national and regional flu activity. Biggerstaff et al. [2] presented the result of a recent FluSight challenge. Reich et al. [3] compared the forecast accuracies of 22 models from five different institutions. Some of the most common approaches in influenza forecasting can be grouped into the following categories [4,5,6]: compartmental models [7,8], agent-based models, direct regression models [9,10] and time series models [11,12,13].

Here we focus on time series models for routinely available public health surveillance data. A state-of-the-art approach to modeling infectious disease counts over time is the endemic-epidemic modeling framework introduced by Held et al. [13] (“HHH”). In the HHH framework, the disease incidence is divided into two components: an endemic component, which models seasonal variations of the background risk, and an epidemic component, which adds autoregressive effects to capture disease spread. Estimation, simulation and visualization of HHH models are implemented in the R package surveillance [14], which has enabled a wide range of epidemiological analyses, including forecasts of infectious disease counts [15]. However, when measuring and forecasting flu activity, the CDC uses the proportion of outpatient visits with ILI rather than absolute surveillance counts, and the total number of outpatient visits is subject to seasonal variation. For this purpose, we borrow the idea of HHH and introduced an endemic-epidemic *beta* model designed for time series of infectious disease proportions. In this model, proportions are modeled using a conditional beta distribution, which naturally keeps the boundedness of proportions and accommodates heteroskedasticity and potential asymmetry of proportion distributions. Likelihood inference is straightforward via the R package betareg [16]. The beta model thus represents a relatively simple and fast approach to forecast proportions.

The purpose of this work is to investigate the usefulness of the endemic-epidemic beta model as a forecasting tool. We benchmark the beta model against several alternative methods with readily available and well-documented implementations in R [17]. These methods are the seasonal autoregressive integrated moving average (SARIMA) model [11], harmonic regression with ARIMA errors [11] and Facebook’s forecasting tool Prophet [18]. Furthermore, kernel conditional density estimation [9], a successful competitor in the FluSight challenge, is also included in our comparison. We apply these models to predict short-term and seasonal flu activity, using similar forecast targets as in the FluSight challenge and relevant to public health. The short-term targets consist of one to four-weeks-ahead forecasts, and the seasonal targets are predictions of the intensity and timing of the seasonal peak. We require all forecasts to be probabilistic, and thus to reflect prediction uncertainty, which is fundamental for decision making [19]. Proper scoring rules are used to evaluate probabilistic forecasts [20].

## 2. Materials and Methods

### 2.1. Data

The U.S. Outpatient Influenza-like Illness Surveillance Network (ILINet) collects weekly information on outpatient visits to health care providers for influenza-like illness (ILI). Here, ILI is defined as “fever (temperature of 100 ${}^{\circ }$F [37.8 ${}^{\circ }$C] or greater) and a cough and/or a sore throat without a known cause other than influenza” [21]. The national weighted influenza-like illness (wILI) index is calculated as the proportion of outpatient visits with ILI reported through ILINet, weighted by state population [21]. It is a standard measure of flu activity in the USA.

CDC publishes the wILI index in their Morbidity and Mortality Weekly Report [21] (MMWR). MMWR weeks start on Sunday and are indexed from 1 to 52 (or 53), where week 1 is the first week with at least four days in the calendar year. In most years, flu activity begins to increase at the beginning of October, peaks between December and February and lasts until May. This period is considered relevant for disease control and roughly corresponds to MMWR week 40 to MMWR week 20 of the following year. In this paper, we index seasons from MMWR week 31 to MMWR week 30 of the following year, so season week 1 corresponds to MMWR week 31.

**Figure 1:**
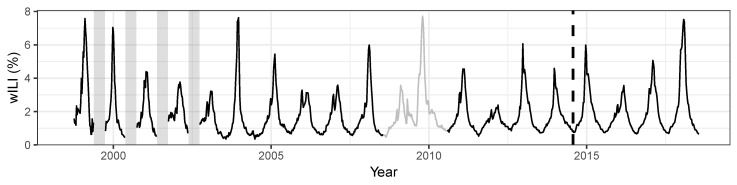
Weekly weighted influenza-like illness (wILI) index in the USA. In early years of data collection, low-season incidence was not recorded. These periods of missing data are indicated with vertical gray bars. The wILI index during the excluded pandemic seasons (2008/2009 and 2009/2010) is shown in gray. The last four seasons (2014/2015 to 2017/2018) after the vertical dashed line are held out as test data.

We used the R package cdcfluview [22] to download CDC’s weekly national wILI data from season 1998/1999 to season 2017/2018 (Figure 1) for our analysis. We excluded the two H1N1 pandemic seasons, 2008/2009 and 2009/2010, since our focus is on seasonal influenza forecasting. The last four seasons are taken as test data to assess forecast performance. ILINet members continuously provide backfill reports for past weeks so the previously reported wILI index may be modified in subsequent weeks [23]. In this paper, however, we ignore the backfill of the wILI data and use the latest available data.

### 2.2. Prediction Targets and Evaluation Criteria

We use prediction targets of the CDC’s FluSight challenges [2] for model comparison. They can be divided into two categories: short-term targets and seasonal targets. The short-term targets are one-week, two-weeks, three-weeks and four-weeks-ahead forecasts of the wILI index. The seasonal targets consist of peak week timing and peak intensity prediction. The peak week of an influenza season is the week which has the highest wILI index of the season, the peak intensity. The CDC formulated these targets to more effectively plan for public health responses to seasonal flu epidemics, including the allocation of treatment resources and mitigation strategies [24].

Gneiting and Katzfuss [19] argued that forecasts should be probabilistic to quantify the uncertainty in a prediction. Probabilistic forecasts are increasingly used in infectious disease epidemiology [25,26], and also requested in the FluSight challenge. The quality of a probabilistic forecast is assessed using proper scoring rules, which are summary measures of predictive performance [19]. For the short-term targets, we calculate the log score [27] and the Dawid-Sebastiani score [28] of the predictive distribution. Denoting the predictive distribution by *F*, the predictive density by *f* and the actual observation by *y*, the log score (LS) is defined as

(1) LS(F,y)=−logf(y).

The Dawid-Sebastiani score (DSS)

(2) $$\mathrm{DSS}\left(F,y\right)=2\log\sigma_{F}+{\left(y-\mu_{F}\right)}^{2}/\sigma_{F}^{2}$$

depends only on the mean $\mu_{F}$ and variance $\sigma_{F}^{2}$ of the predictive distribution. We also report the absolute error (AE)

(3) $$\mathrm{AE}\left(y,\hat{y}\right)=\left|y-\hat{y}\right|,$$

which is the distance between the actual observation *y* and the point forecast $\hat{y}$.

For seasonal targets, we simulate 10,000 trajectories starting from each week between season week 11 and season week 41 to season week 42. For each starting week, we obtain the empirical distribution of peak timing ${\hat{F}}_{Time}$ from those simulated trajectories. Then, the log score of peak timing prediction for one starting week is calculated as $-\log({\hat{F}}_{Time}\left(w\right)-{\hat{F}}_{Time}\left(w-1\right))$, where *w* is the actual peak week. For peak intensity prediction, we calculate the log score on a binned proportion scale with bins [0,0.5%), [0.5%,1%),⋯, [13%,100%]. For each starting week, we obtain the empirical distribution of the binned peak week intensity ${\hat{F}}_{Int}$ from the simulated trajectories. The log score for peak intensity prediction is calculated as $-\log({\hat{F}}_{Int}\left(I\right)-{\hat{F}}_{Int}\left(I-1\right))$, where *I* is the index of the bin where the actual peak intensity lies.

All scores are thus defined as negatively oriented penalties that we wish to minimize: the smaller the score, the better the forecast. We report average scores over the whole test period and over subsets of the test period. We also repeat the calculation of the maximum log score by Ray et al. [9] as a measure of worst-case performance. This helps to compare models with similar overall performances, as measured by the mean log score. The differences between mean log scores are statistically evaluated via permutation tests for paired observations [29]. Note that the log score used in this paper is not directly comparable with the log score used in the FluSight challenge. The CDC calculates the log score of multiple bins surrounding the actual observation, which is, however, not a proper score [30].

### 2.3. Endemic-Epidemic Beta Model

We propose an extension of the HHH framework for infectious disease proportions [31]. Let $X_{t}$ denote the proportion of newly infected individuals by a certain disease at time t=1,…,T. The model assumes $X_{t}$ to follow a beta distribution with mean $\mu_{t}$ and precision $\phi_{t}$ conditional on past observations,

(4) $$X_{t}|F_{t-1}∼\mathrm{Beta}\left(\mu_{t},\phi_{t}\right),$$

(5) $$g\left(\mu_{t}\right)=\nu_{t}+\sum_{k=1}^{p}\beta_{k}g\left(X_{t-k}\right),$$

where $g\left(x\right)=\log\left(\frac{x}{1-x}\right)$ is the logit link function, and $F_{t-1}=\sigma \left(X_{1},\cdots ,X_{t-1}\right)$. The transformed conditional mean $g(\mu_{t})$ is decomposed into an endemic component $\nu_{t}$ and an epidemic component $\sum_{k=1}^{p}\beta_{k}g\left(X_{t-k}\right)$. The endemic component is modeled as a linear predictor of covariates $z_{t}^{\left(\nu \right)}$,

(6) $$\nu_{t}=\alpha^{\left(\nu \right)}+\beta^{{\left(\nu \right)}^{⊤}}z_{t}^{\left(\nu \right)}.$$

The precision parameter $\phi_{t}$ can also be time-varying and depend on covariates $z_{t}^{\left(\phi \right)}$ with

(7) $$\log\left(\phi_{t}\right)=\alpha^{\left(\phi \right)}+\beta^{{\left(\phi \right)}^{⊤}}z_{t}^{\left(\phi \right)}.$$

The endemic component $\nu_{t}$ and the precision parameter $\phi_{t}$ could be modeled by harmonic regression; e.g., $z_{t}^{\left(\phi \right)}={\left(\sin\left(\omega t\right),\cos\left(\omega t\right),\cdots ,\sin\left(S_{\phi }\cdot \omega t\right),\cos\left(S_{\phi }\cdot \omega t\right)\right)}^{⊤}$, where $S_{\phi }$ denotes the number of harmonics and $\omega =\frac{2\pi }{52}$ for weekly data [32]. Potential holiday effects could be included via additional dummy variables.

We denote the above model by Beta(*p*), where *p* is the maximum order of the autoregressive terms. It can be regarded as a distributional regression model, where the exponentiated $\beta^{\left(\nu \right)}$ and $\beta_{k}$ parameters can be interpreted as odds ratios [31]. Parameter estimation can be carried out by (conditional) maximum likelihood using the R package betareg [16].

AIC is a useful criterion for model selection if prediction is the exclusive purpose [33]. However, AIC causes overfitting when the sample size is small or the number of parameters is relatively large. We thus use AICc for model selection [34].

### 2.4. Baseline Models

Five baseline models are considered for comparison. The first baseline model is a SARIMA model fitted on the logit-transformed proportion time series. We use the auto.arima function from the R package forecast [11] to determine the order of the SARIMA model by a stepwise procedure on the training data.

The second baseline model is a harmonic regression model with ARIMA errors, or ARIMA for short, with regressors containing holiday effects via dummy variables. The ARIMA model is fitted on the logit-transformed proportions, and model selection is performed on the training data. The number of harmonics is chosen by AICc, and the order of the ARIMA part is chosen by auto.arima.

Ray et al. [9] proposed an approach to generate predictions of disease incidence by combining kernel conditional density estimation (KCDE) and copulas. For each prediction horizon, predictive distributions are estimated by KCDE, and copulas tie these predictive distributions into joint distributions. They evaluated different KCDE model specifications, and in most cases of their application, the KCDE model with periodic kernel components and a full bandwidth matrix had a better forecast performance than the other model specifications. Accordingly, we use that as our third baseline model, following the implementation provided by the authors.

Facebook’s Core Data Science team developed the automatic forecasting procedure Prophet and implemented it in the R package prophet [18]. Their model corresponds to a Bayesian harmonic regression model with trend changepoints and holiday effects. Since Prophet assumes Gaussian errors, we fit the proportion time series on the logit scale.

The fifth baseline model is a naive approach: for each test season we estimate a logitnormal distribution of the wILI based on the same calendar week in the previous seasons.

For seasonal targets we consider an additional reference approach which assigns equal probabilities to all possible outcomes. For prediction of peak timing, equal probabilities are assigned to season weeks 10 to 42. For peak intensity prediction, equal probabilities are assigned to all 27 bins.

The beta model, ARIMA model, SARIMA model and KCDE model are dynamic models, and the Prophet model, naive approach and equal-bin approach are non-dynamic models.

## 3. Results

### 3.1. Model Selection

**Figure 2:**
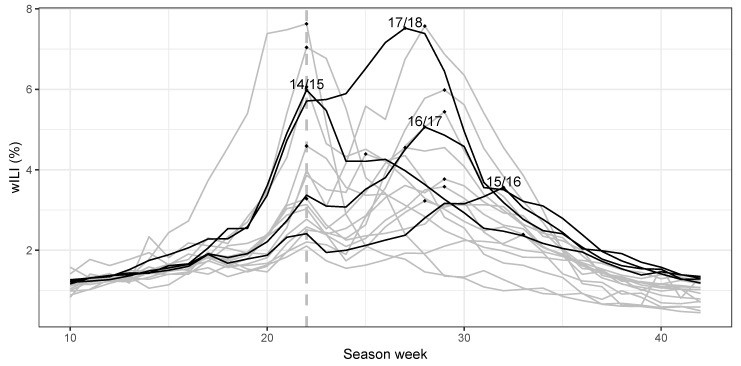
Weighted influenza-like illness (wILI) in the USA for flu seasons 1998 through 2017. The pandemic seasons are excluded. Season week 22 is indicated with a vertical dashed line, where a peak or secondary peak occurs in most seasons. Data in the training and test seasons are in gray and black, respectively. The peak of each season is indicated by a dot.

Our analysis is based on the national wILI data in the USA from season 1998/1999 to season 2017/2018. In most seasons, a peak or secondary peak occurred in season week 22, which corresponds to MMWR week 52 (Figure 2). This (intermediate) peak can be explained by the fact that during the winter holidays, patients tend not to visit the doctor for less severe illness, thereby reducing the number of non-ILI visits and consequently increasing wILI [6]. During the winter holidays, the transmission of ILI is hampered due to a reduction of work and school contacts [35]. Then, the number of ILI visits decreases towards the end of the winter holidays, and wILI drops during season week 23 [6]. To capture this pattern in our models, we included two dummy variables $x_{t}$ and $y_{t}$ for season weeks 22 and 23, respectively, in the covariate vector $z_{t}^{\left(\nu \right)}$.

As Figure 1 shows no time trend or increasing/decreasing variation of wILI from 1998 to 2018, we omitted a time trend in the endemic part of the beta model. The number of harmonics $S_{\nu }$ and $S_{\phi }$, and the order of autoregressive terms *p*, were jointly chosen by AICc using the training data. This procedure resulted in a beta model with p=4, $S_{\nu }=3$ and $S_{\phi }=3$. Moreover, we included the beta model with p=1, $S_{\nu }=3$ and $S_{\phi }=4$, which had the best AICc among the beta models with one autoregressive lag. For the SARIMA and ARIMA models, AICc-based model selection using the training data resulted in SARIMA(1,0,0)(1,1,0)[52] and ARIMA(5,1,0) with S=4 harmonics, respectively. The final number of parameters for each of these models is given in Table 1, and it ranges from 3 (SARIMA) to 20 (Beta(4)).

For beta and (S)ARIMA, training data were used to determine the structure of the model (e.g., number of autoregressive terms and harmonics). In the test period, parameters were reestimated given the observations until the specified time point, while the structure of the model was kept. For KCDE, we implemented the approach of Ray et al. [9], which excludes reestimation, and thus considerably reduces the total run time. Initial KCDE fitting took around 45 min per prediction horizon. Note that seasonal forecasting with KCDE involves copula estimation, which considerably increases the number of parameters (720 in total for this application) and the run time. The structure and parameters of the Prophet model were both updated throughout the test period, since Prophet updates its automatic change points when more observations become available.

### 3.2. Short-Term Targets

The short-term performance is summarized in Table 1, averaged over all one to four-weeks-ahead forecasts. We consider two kinds of subsets in this comparison. The “all weeks” subset averages the log scores and the Dawid-Sebastiani scores over the whole test period. The “weeks 40–20” subset gives the average scores over the high incidence periods only, which are of particular interest for disease control.

For the “all weeks” subset, KCDE has the best Dawid-Sebastiani score, the lowest maximum log score and the lowest absolute error. The Beta(4) model has the best mean log score and second-best forecast performance in terms of maximum log score, absolute error and Dawid-Sebastiani score. Forecasting with Beta(4) is considerably faster than with KCDE and has fewer parameters to estimate. Then follow Beta(1), ARIMA and SARIMA. The Prophet and naive methods produce the worst short-term forecasts. Similar rankings are obtained when considering average scores over the MMWR weeks 40–20 only. Beta(4) outperforms Beta(1) for both subsets. To address statistical significance, we ran pairwise Monte Carlo permutation tests for the log scores using Beta(4) as the reference. In both subsets, there is no evidence for a difference between Beta(4) and KCDE. ARIMA, SARIMA, Prophet and naive perform significantly worse.

A more detailed view stratified by prediction horizon is given in Table 2. The forecast performance of Beta(4) decreases over prediction horizons. For the one-week-ahead forecasts, Beta(4) outperforms all other models in terms of mean log score and Dawid-Sebastiani score for both subsets. For higher prediction horizons and in both subsets, KCDE has the best or close to the best mean log score and Dawid-Sebastiani score, while Beta(4) is always the second-best, except that for the two-weeks-ahead forecasts, Beta(4) has the best mean log score.

**Figure 3:**
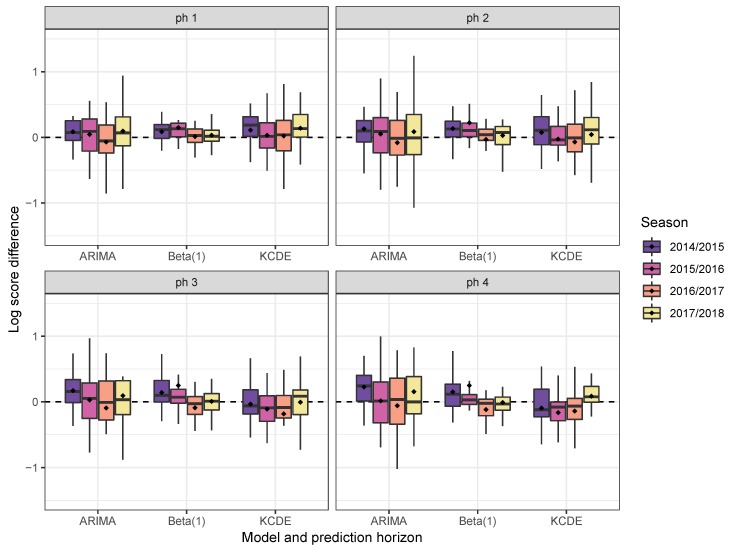
Boxplots of the weekly log-score differences of Beta(4) vs. Beta(1), KCDE and ARIMA, respectively, for prediction horizons of one to four weeks in the “weeks 40–20” subset of the test period, stratified by season. Positive values indicate superiority of Beta(4). The mean is marked with dots.

Boxplots of the log-score differences of Beta(4) vs. Beta(1), KCDE and ARIMA, respectively, stratified by test season (weeks 40–20 only) and prediction horizon, are shown in Figure 3. Not one of the methods uniformly outperforms another. For higher prediction horizons of three or four weeks, KCDE outperforms Beta(4) (on average) in all but the last test season.

### 3.3. Seasonal Targets

For seasonal targets, we average model performance over two alternative periods: either “all weeks” of the test period or only the weeks before the actual peak of the given season (“before peak”). In the FluSight challenges, the forecast performance of the "all week” subset is evaluated, while in practice, peak predictions before the actual peak are more meaningful to health officials [30].

The model performance for seasonal targets is summarized in Table 3. In terms of the average log score, the equal-bin approach has the worst forecast performance for both seasonal targets and in both subsets. For peak intensity prediction and in the “all weeks” subset, KCDE has the best performance in terms of the average log score. Naive has the second-best performance, followed by Beta(1), Beta(4), SARIMA, ARIMA and Prophet. The ranking of the models is the same in the “before peak” subset. However, considering the results from the pairwise permutation tests, only ARIMA, SARIMA, Prophet and the equal-bin approach perform significantly worse than KCDE. There is no evidence that Beta(1) or the naive approach perform worse than KCDE.

For peak timing prediction, KCDE has the best mean log score in the “all weeks” subset, followed by Prophet, ARIMA, naive, Beta(4), SARIMA and Beta(1). In the “before peak” subset, ARIMA performs best and KCDE follows closely behind, while the rankings of the other models are similar. According to the permutation tests, Beta(1), SARIMA and the equal-bin approach predict peak timing significantly worse than KCDE.

**Figure 4:**
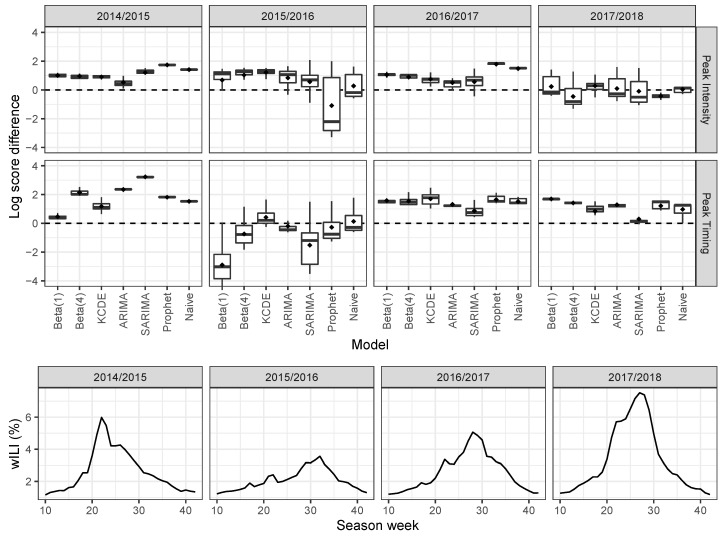
Each boxplot (top) summarizes predictions made by a model in the subset “before peak.” The vertical axis is the difference in log score between the given model and the equal-bin approach. Positive values favor the model over the equal-bin approach. The mean is indicated by dots. The bottom plot shows the epidemic curve for each season.

We now investigate the forecast performance in the subset “before peak" for each of the test seasons separately. Figure 4 shows boxplots of log-score differences by season, using the equal-bin approach as a reference. For the 2014/2015 season, all models show better forecast performance than equal bin in both peak intensity and timing prediction. Prophet has the best peak intensity prediction, and SARIMA has the best peak timing prediction. This season evolved with a relatively standard epidemic curve (see also Figure 2).

In season 2015/2016, the peak intensity was lower and the peak occurred later than in most training seasons. For peak intensity prediction, KCDE scores best, and only Prophet is worse than equal bin. Peak timing prediction is more difficult in this season. KCDE ranks first and naive second, and only these two approaches are better than equal bin.

Season 2016/2017 was again a relatively normal season. All models provide better predictions than equal bin for both targets. Two non-dynamic models, Prophet and naive, have better peak intensity predictions than other models. For peak timing prediction, KCDE has the best score and is closely followed by prophet and naive.

Season 2017/2018 had a normal peak timing, but a relatively high peak intensity. KCDE has the best peak intensity prediction, followed by Beta(1), ARIMA and naive. Other models have worse scores than equal bin. All models have better peak timing prediction than equal bin, and Beta(1) ranks first.

Over all test seasons and both seasonal targets, only KCDE and naive have consistently better forecast performances than equal bin. Additionally, no model is consistently better than naive.

## 4. Discussion

In this paper, the performances for both short-term and peak forecasts of a new beta model for time series of infectious disease proportions were compared with some common alternatives and with the recently proposed KCDE approach.

Regarding short-term prediction, the Beta(4) model produced the best one-week-ahead forecasts in terms of the mean log score and Dawid-Sebastiani score. The performance of short-term forecasts of the beta model was improved by increasing the number of autoregressive lags from one to four. KCDE performed better for higher prediction horizons, and Beta(4) ranked second. The KCDE approach is more complex, and forecasting took more than four hours, whereas Beta(4) needed 2.6 min. Prophet and the naive approach did not provide useful short-term forecasts.

Regarding peak prediction, the relative performance of the different models varied by season. For peak intensity, the two non-dynamic models, Prophet and naive, ranked best in normal seasons (seasons 2014/2015 and 2016/2017), while KCDE scored best in seasons with unusual epidemic curves (seasons 2015/2016 and 2017/2018). For peak timing, KCDE performed best in season 2015/2016, when the wILI proportion peaked relatively late. In the other seasons, SARIMA, KCDE or Beta(1) ranked first. In short, forecasts from KCDE were most robust with respect to the epidemic curve, and Prophet produced the best peak intensity forecasts in normal seasons.

In comparison to the alternative models, the beta model is competitive, especially in short-term forecasting, and has a relatively simple model structure and short run time. Furthermore, incorporation of covariates in the beta model is a straightforward extension. For example, time series data from social media such as Twitter or Wikipedia search queries could improve forecasts [23]. Our comparison here was limited to models with no external data.

We only included readily available and well-documented models, and KCDE, in our model comparison. Some other competitive models presented in the FluSight challenge are the delta density method [6], the empirical Bayes framework [10] and the dynamic Bayesian model [36]. Some time series models for proportions are also applicable to flu activity forecasting, such as the βARMA model [37] and the marginal beta regression time series model [38]. Makridakis et al. [39] evaluated the performances of statistical and machine learning forecasting methods using a large monthly time series and observed that machine learning methods are dominated by classical statistical methods for both long-term and short-term forecasts. Thus, we did not include machine learning methods in our comparison.

In this analysis, we generated forecasts retrospectively based on the latest available wILI data. Forecasts in real-time suffer from reporting delays. ILINet members continuously provide backfill reports for past weeks, and the CDC modifies reported data accordingly. Models accounting for such backfilling [6] or incorporating internet-based nowcasting [23] can improve forecast performance in real-time.

Other promising developments are multi-model ensembles [3,30]. As discussed above, peak forecasts from KCDE were most robust over different seasons, while Prophet had the best peak intensity forecasts in standard seasons. More generally, all models try to capture seasonality somehow, and are more or less sensitive to deviations from the expected pattern. Beta, ARIMA and Prophet employ harmonic regression; KCDE uses a periodic kernel; and SARIMA uses seasonal differencing. Multi-model ensembles with adaptive weights should be able to combine the advantages of these different approaches. For example, when predicting peak intensity, more weight could be assigned to Prophet if available observations were to indicate a standard season and vice versa.

## 5. Conclusions

In this paper, we compared the forecast performance of a new beta model for time series of infectious disease proportions with readily available baseline models. In conclusion, the beta model was competitive in short-term forecasting with a simple structure and short run time, and KCDE was the most robust for peak forecasts. Multi-model ensembles with adaptive weights should be considered, especially for seasonal forecasts. Code and data to reproduce our analysis are available online at https://github.com/Junyi-L/USfluIndex.

## Figures and Tables

**Table 1:** Model performance in terms of log score (LS), Dawid-Sebastiani score (DSS) and absolute error (AE), averaged over all short-term targets. Ranks are shown in brackets. The “all weeks” group shows average scores over the whole test period (n = 836), whereas the “weeks 40–20” group shows averages over the high incidence periods only (n = 532). The total run times for estimation, reestimation and forecasting at all time points for all prediction horizons are given in minutes. For KCDE, the total run time does not include reestimation. The last column gives the number of estimated parameters. The Monte Carlo p-values for differences in mean log scores are based on 9999 random permutations, comparing each model against Beta(4).

Model	Subset	LS	*p*-Value	maxLS	DSS	AE	Time	npar
Beta(1)	All weeks	–0.11 (3)	0.1862	5.59 (7)	–2.02 (3)	0.25 (3)	2.91 (3)	19 (3)
Beta(4)		–0.12 (1)		4.34 (2)	–2.07 (2)	0.25 (2)	2.64 (2)	20 (4)
KCDE		–0.12 (2)	0.8076	4.08 (1)	–2.29 (1)	0.24 (1)	266.63 (7)	28 (5)
ARIMA		–0.02 (4)	0.0001	5.24 (5)	–1.81 (4)	0.28 (5)	6.22 (4)	16 (2)
SARIMA		0.04 (5)	0.0001	4.92 (3)	–1.69 (5)	0.27 (4)	110.37 (6)	3 (1)
Prophet		0.48 (7)	0.0001	5.04 (4)	–0.75 (7)	0.44 (6)	11.75 (5)	50 (6)
Naive		0.42 (6)	0.0001	5.29 (6)	–1.13 (6)	0.46 (7)	0.07 (1)	106 (7)
Beta(1)	weeks 40–20	0.43 (4)	0.0001	5.59 (7)	–0.94 (4)	0.37 (3)		
Beta(4)		0.35 (2)		4.34 (2)	–1.10 (2)	0.35 (2)		
KCDE		0.33 (1)	0.3160	4.08 (1)	–1.28 (1)	0.34 (1)		
ARIMA		0.41 (3)	0.0186	5.24 (5)	–0.98 (3)	0.39 (4)		
SARIMA		0.50 (5)	0.0001	4.92 (3)	–0.77 (5)	0.39 (5)		
Prophet		0.97 (7)	0.0001	5.04 (4)	0.19 (7)	0.64 (6)		
Naive		0.97 (6)	0.0001	5.29 (6)	0.02 (6)	0.67 (7)		

**Table 2:** Model performance in terms of log score (LS) and Dawid-Sebastiani score (DSS), averaged by prediction horizon (ph) of one-to-four weeks ahead. Ranks are shown in brackets. The “all weeks” group shows average scores over the whole test period, whereas the “weeks 40–20” group shows averages over the high incidence periods only.

Model	Subset	ph1		ph2		ph3		ph4	
		LS	DSS	LS	DSS	LS	DSS	LS	DSS
Beta(1)	All weeks	–0.53 (2)	–2.90 (2)	–0.15 (3)	–2.10 (3)	0.06 (3)	–1.68 (3)	0.18 (2)	–1.41 (3)
Beta(4)		–0.55 (1)	–2.96 (1)	–0.18 (1)	–2.19 (2)	0.05 (2)	–1.71 (2)	0.19 (3)	–1.42 (2)
KCDE		–0.42 (4)	–2.89 (3)	–0.16 (2)	–2.33 (1)	–0.02 (1)	–2.11 (1)	0.12 (1)	–1.83 (1)
ARIMA		–0.47 (3)	–2.76 (4)	–0.09 (4)	–1.96 (4)	0.15 (4)	–1.46 (4)	0.33 (4)	–1.07 (5)
SARIMA		–0.39 (5)	–2.61 (5)	–0.02 (5)	–1.82 (5)	0.21 (5)	–1.34 (5)	0.36 (5)	–0.98 (6)
Prophet		0.46 (7)	–0.78 (7)	0.47 (7)	–0.76 (7)	0.48 (7)	–0.73 (7)	0.49 (7)	–0.72 (7)
Naive		0.42 (6)	–1.13 (6)	0.42 (6)	–1.13 (6)	0.42 (6)	–1.13 (6)	0.42 (6)	–1.13 (4)
Beta(1)	weeks 40–20	–0.03 (3)	–1.91 (4)	0.38 (4)	–1.05 (4)	0.62 (4)	–0.56 (4)	0.76 (3)	–0.24 (3)
Beta(4)		–0.10 (1)	–2.07 (1)	0.29 (1)	–1.24 (2)	0.54 (2)	–0.72 (2)	0.69 (2)	–0.39 (2)
KCDE		–0.03 (4)	–2.05 (2)	0.30 (2)	–1.33 (1)	0.46 (1)	–1.05 (1)	0.61 (1)	–0.70 (1)
ARIMA		–0.06 (2)	–1.95 (3)	0.34 (3)	–1.12 (3)	0.59 (3)	–0.63 (3)	0.77 (4)	–0.23 (4)
SARIMA		0.00 (5)	–1.82 (5)	0.43 (5)	–0.94 (5)	0.70 (5)	–0.37 (5)	0.88 (5)	0.07 (6)
Prophet		0.95 (6)	0.14 (7)	0.97 (6)	0.17 (7)	0.98 (7)	0.21 (7)	0.99 (7)	0.23 (7)
Naive		0.97 (7)	0.02 (6)	0.97 (7)	0.02 (6)	0.97 (6)	0.02 (6)	0.97 (6)	0.02 (5)

**Table 3:** Summaries of model performances for predictions of peak intensity and peak timing. The “all weeks” group shows the average log scores (LS) and maximum log scores (maxLS) over all predictions in the test period (n = 124). The “before peak” group summarizes the log scores for predictions in weeks before the actual peak of the given season (n = 63). The Monte Carlo *p*-values for differences in mean log scores are based on 9999 random permutations, comparing each model against KCDE.

Model	Subset	Peak Intensity			Peak Timing		
		LS	*p*-Value	maxLS	LS	*p*-Value	maxLS
Beta(1)	All weeks	1.46 (3)	0.2647	5.26 (7)	1.99 (7)	0.0001	8.11 (8)
Beta(4)		1.51 (4)	0.0305	4.60 (6)	1.47 (5)	0.4714	5.32 (6)
KCDE		1.41 (1)		4.03 (3)	1.43 (1)		4.87 (5)
ARIMA		1.59 (6)	0.0001	4.06 (4)	1.44 (3)	0.8248	4.12 (3)
SARIMA		1.57 (5)	0.0053	4.34 (5)	1.78 (6)	0.0017	7.01 (7)
Prophet		1.68 (7)	0.0338	6.57 (8)	1.44 (2)	0.8870	4.76 (4)
Naive		1.46 (2)	0.4184	3.87 (2)	1.46 (4)	0.5251	4.10 (2)
Equal bin		3.30 (8)	0.0001	3.30 (1)	3.50 (8)	0.0001	3.50 (1)
Beta(1)	Before peak	1.69 (3)	0.2971	5.26 (7)	2.31 (7)	0.0003	8.11 (8)
Beta(4)		1.76 (4)	0.0784	4.60 (6)	1.71 (5)	0.6756	5.32 (6)
KCDE		1.63 (1)		4.03 (3)	1.65 (2)		4.87 (5)
ARIMA		1.83 (6)	0.0006	4.06 (4)	1.64 (1)	0.6116	4.12 (3)
SARIMA		1.83 (5)	0.0058	4.34 (5)	2.05 (6)	0.0033	7.01 (7)
Prophet		1.96 (7)	0.0178	6.57 (8)	1.67 (3)	0.5846	4.76 (4)
Naive		1.69 (2)	0.3297	3.87 (2)	1.68 (4)	0.4014	4.10 (2)
Equal bin		3.30 (8)	0.0001	3.30 (1)	3.50 (8)	0.0001	3.50 (1)
